# Measuring successful aging: an exploratory factor analysis of the InCHIANTI Study into different health domains

**DOI:** 10.18632/aging.101957

**Published:** 2019-05-24

**Authors:** Sarah Mount, Luigi Ferrucci, Anke Wesselius, Maurice P. Zeegers, Annemie MWJ Schols

**Affiliations:** 1 Department of Respiratory Medicine, NUTRIM School of Nutrition and Translational Research in Metabolism, Maastricht University Medical Centre, Maastricht, the Netherlands; 2 Longitudinal Studies Section, Translational Gerontology Branch, National Institute on Aging, National Institutes of Health, Baltimore, MD 21224, USA; 3 Department of Complex Genetics, NUTRIM School of Nutrition and Translational Research in Metabolism, Maastricht University Medical Centre, Maastricht, the Netherlands

**Keywords:** successful aging, healthy aging, model, index, active aging

## Abstract

Advocating continued health into old age, so called successful aging, is a growing public health goal. However, the development of tools to measure aging is limited by the lack of appropriate outcome measures, and operational definitions of successful aging. Using exploratory factor analysis, we attempted to identify distinguishable health domains with representative variables of physical function, cognitive status, social interactions, psychological status, blood biomarkers, disease history, and socioeconomic status from the InCHIANTI study. We then used logistic and mixed effect regression models to determine whether the resulting domains predicted outcomes of successful aging over a nine-year follow-up. A four-domain health model was identified: neuro-sensory function, muscle function, cardio-metabolic function and adiposity. After adjustment for age and gender, all domains contributed to the prediction of walking speed (R^2^=0.73). Only the muscle function domain predicted dependency (R^2^=0.50). None of the domains were a strong, significant predictor of self-rated health (R^2^=0.18) and emotional vitality (R^2^=0.23). Cross-sectional findings were essentially replicated in the longitudinal analysis extended to nine-year follow-up. Our results suggest a multi-domain health model can predict objective but not subjective measures of successful aging.

## Introduction

The number of old and very old adults (aged 65 and over, and 80 and over respectively) is rapidly rising in all European countries, and represents a progressively growing percentage of the general population [1]. At the same time, the proportion of working aged individuals is declining [2]. These changes in the population pyramid, as well as increasing life expectancy, is challenging the stability of health and social care systems [3]. Therefore, advocating strategies that promote health into old age and maximise *successful aging* is a growing public health goal.

Biological aging varies markedly between individuals [4], and this disparity between individuals only grows with age [5]. Although partially genetically determined, 75% of human longevity is believed to be determined by modifiable factors including diet, lifestyle and socioeconomic status [4]. In order to understand whether any intervention aimed at promoting healthy aging is effective, a benchmark for the assessment of healthy aging is needed. Therefore, the development of tools to measure successful aging, and to timely identify the early stages of health impairment, has become a research priority [6]. Developing such tools however, is a challenge, as aging is a complex process and it is unlikely that a single measure will be able to track the aging trajectory, particularly early in life, when disease symptoms and functional limitations are still rare [6]. Furthermore, testing the validity of tools to measure healthy aging is complicated due to the lack of an agreed upon definition of healthy aging [6], as well as discrepancies in the terminology describing this concept.

As of 2010, 29 different definitions of successful aging have appeared in the literature [7]; Michel & Sadana summarised the recent conceptualisations of aging [8], and more recently, a citation network analysis identified 1146 publications related to successful aging [9]. Despite extensive discussion and substantial analytical work done on defining successful aging, many authors agree that an objective, robust measure of healthy aging is difficult to develop. This is because at the individual level, successful aging depends heavily on perspective of the observer, and different individuals may value different aspects of their life [9]. Subjective definitions tend to include themes such as the attainment or maintenance of goals, positive attitudes, attainment of social milestones and connectedness, whereas objective measures emphasise lack of disease and preserved functional status [7]. Yet, until a reliable measure of biological aging can be developed, measures based on the aggregation of phenotypes, functional status, as well as subjective goals remain the best choice.

Previously defined key aspects of healthy aging include physical and mental health as represented by walking speed [10], dependency risk [11,12], emotional vitality [13] and self-rated health [14,15]. Walking speed is a complex movement which integrates circulatory, respiratory, skeletal, muscular and nervous systems [16]; in older persons, it is a key indicator of physical health and a strong predictor of all-cause, cardiovascular, and other-cause mortality [17–20]. Dependency risk (deficits in the activities of daily living, ADL’s), is strongly associated with severity of health status deterioration and a strong predictor of healthcare utilisation [21]. Emotional vitality is a subjective measure that summarises aspects of mental health, mood, psychological resilience and personal mastery [13], all of which reflect the ability to adapt to changing personal circumstances [22], physical changes [13], and might contribute to the ability to find continued meaning in life. Lastly, self-rated health is a measure used widely in public health as an all-encompassing measure of health status [23], thought to reflect brain-body communication [24].

Due to the multidimensional nature of aging, and age-related pathologies, assessing healthy aging by combining information across many different measurements makes sense. Of the many proposed measurement tools, that at least in theory, assess health and wellbeing in older persons, most focus on disease and disability, which are only partial component of the multifaceted readouts of healthy aging [25]. The problem with these tools is that they can easily distinguish the least healthy but not the healthiest individuals in the population [26]. This is because diseases and disabilities only become manifest when compensatory strategies are exhausted. Lara *et. al.* proposed that this problem could be addressed by operationally defining the Healthy Aging Phenotype (HAP) [25], a panel of measures which change with age, are susceptible to lifestyle interventions and that can be classified in few meaningful domains. Clustering variables into domains may not only ease the interpretation of complex health data, it can also provide some clues of the underlying mechanism that affect the “healthy” condition. Furthermore, if the domains are identified using empirical methods, such as exploratory factory analysis, sub-scores can be developed that can capture changes in health status. Almost surprisingly, the development and use of such empirical methods have been limited to only a few studies [27,28].

**Figure 1:**
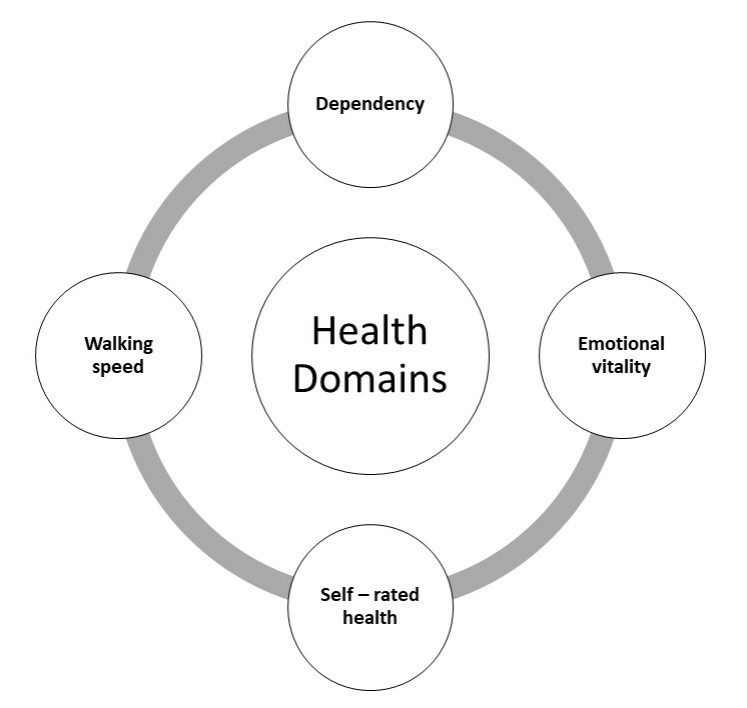
**Theoretical model of successful aging.**

Using data from the InCHIANTI study, we aimed to identify distinguishable variable clusters (hereafter referred to as domains) that have face-validity for healthy aging. We hypothesise that meaningful domains can be derived from the data, and that they are predictive of key aspects of successful aging (Figure 1).

## RESULTS

### Exploratory factor analysis

**Figure 2:**
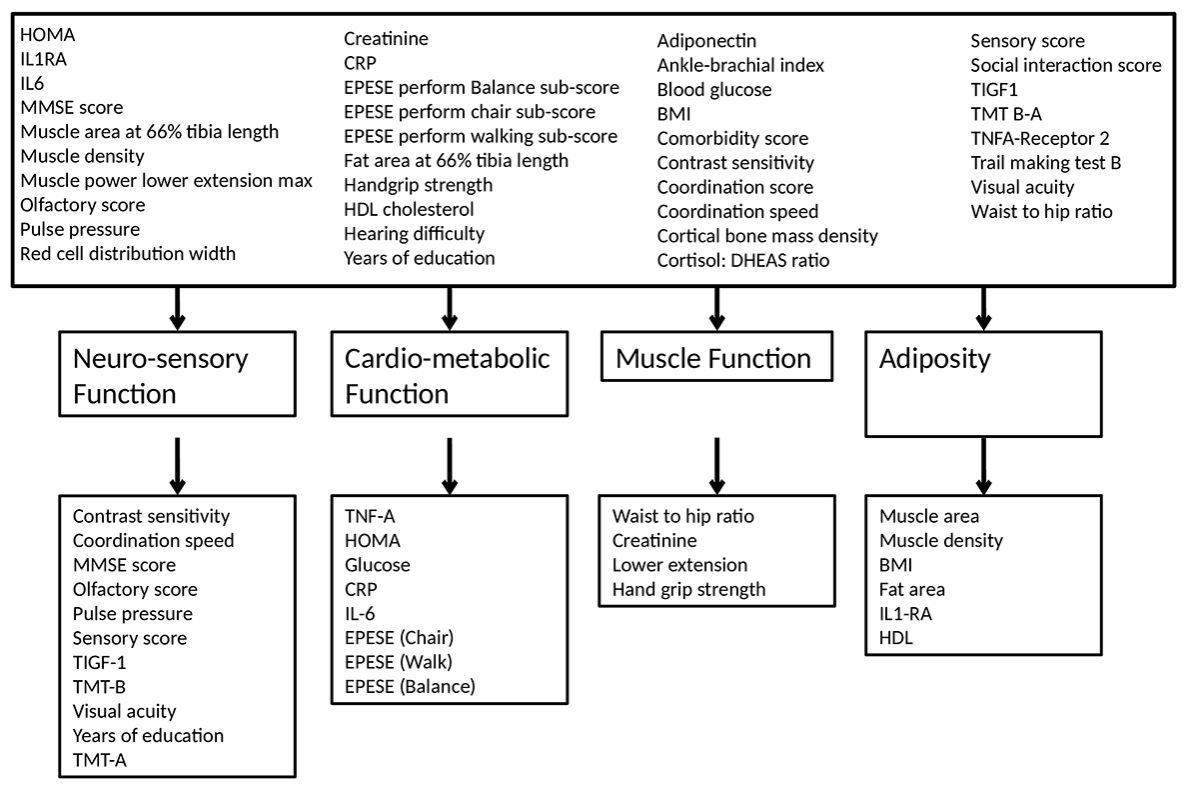
**Extracted factor loading.** Four factors (domains) named: Neuro-sensory function, Cardio-metabolic function, Muscle function and Adiposity.

Table 1 shows the factors resulting from the oblique rotation (Table 1). All factors were used in further analysis (refer to Supplementary Table S1 for the orthogonally rotated factor loadings). Using the Eigen value criteria (Table 1) as well as visual inspection of the scree plot (Supplementary Figure S1 ) four factors were identified defined as neuro-sensory function, cardio-metabolic function, muscle function and adiposity and were retained in the model (Table 1, and Figure 2). The factor set was then subjected to the KMO test, which had an overall value of 0.8447, indicating sufficient sampling adequacy.

**Table 1:** Oblique rotated factor loadings.

**Variable**	**Factor 1**	**Factor 2**	**Factor 3**	**Factor 4**	**Uniqueness**
**Adiponectin**					0.8279
Fat area at 66% tibia length			0.4029	-0.8131	0.4144
Muscle area at 66% tibia length		0.3521	0.5086		0.6889
Muscle density	0.3936		-0.3447		0.6768
TNFA-Receptor 2		-0.3775			0.6192
HOMA		-0.3022	0.4763		0.6636
Blood glucose		-0.3355			0.7463
Creatinine				-0.5614	0.6918
**Red cell distribution width**					0.8993
Pulse Pressure	-0.4467				0.7458
Waist to hip ratio				0.4770	0.5632
EPESE perform walking sub-score		0.4925			0.6374
EPESE perform chair sub-score		0.4801			0.5802
EPESE perform Balance sub-score		0.5554			0.6092
**Coordination score**					0.9651
Coordination speed	0.6998				0.4397
**Comorbidity score**					0.7658
Muscle power lower extension max R side	0.5839			0.4267	0.3072
Trail making test B	-0.7686				0.4265
Years of education	0.7623				0.5005
**Hearing difficulty**					0.8857
IL6		-0.4676			0.7674
CRP		-0.3077			0.8350
IL1RA			0.4726		0.7427
**Cortisol: DHEAS ratio**					0.9549
**Ankle-brachial index**					0.9402
**Cortical bone mass density**					0.8337
HDL cholesterol			-0.4382		0.6506
TIGF1	0.4558				0.8022
Olfactory score	0.3925				0.7618
Sensory score	0.6311				0.5171
**Social interaction score**					0.8937
Handgrip strength	0.4989			0.4700	0.4041
BMI			0.8377	-0.3144	0.3811
Visual acuity	0.5421				0.6950
Contrast sensitivity	0.4651				0.6863
MMSE score	0.6832				0.5785

### Reliability testing

Neuro-sensory function, cardio-metabolic function, muscle function and adiposity variable clusters had, respectively, Cronbach’s alpha coefficients of 0.78, 0.74, 0.65 and 0.55. Each of these factors remained stable when retested to derive the alpha value.

### Factor scoring

The regression derived variable weights are presented in Supplementary Tables S2-S5.

### Prediction models

Baseline: All domains were significant independent determinants of *walking speed* with a R^2^ value of 0.78 (MSE 0.15) (Table 2). Next to male gender, only muscle function was predictive of *dependency* with a R^2^ of 0.50 (Table 3). Predictive value for baseline *self-rated health* and *emotional vitality* models was low (R^2^= 0.23 and 0.17 respectively (Tables 4-5)), but it is of note that muscle function, neuro-sensory function and cardio-metabolic function contributed significantly to *self-rated health,* while only cardio-metabolic function contributed significantly to *emotional vitality*. An overview of the results is presented in Table 6.

**Table 2:** Walking speed baseline model predictions.

**Walking speed**	**Coef.**	**Std. Err.**	**t**	**P>|t|**	**95% Conf. Interval**	
					
Gender	-0.038	0.021	-1.850	0.065	-0.079	0.002
Age at baseline	-0.004	0.001	-6.090	0.000	-0.005	-0.003
Adiposity	-0.041	0.008	-5.110	0.000	-0.057	-0.026
Muscle function	0.140	0.014	10.050	0.000	0.112	0.167
Cardio-metabolic function	0.211	0.015	14.460	0.000	0.183	0.240
Neuro-sensory function	0.053	0.011	4.700	0.000	0.031	0.075
constant	1.536	0.043	36.120	0.000	1.453	1.620
Model information						
						
Observations	598	R^2^ adj.	0.779			
p Model	0.000	Root MSE	0.147			
R^2^	0.781	F(6, 691)	351.950			


**Table 3:** Dependency baseline model predictions.

**Dependency**	**Coef.**	**Std. Err.**	**z**	**P>|z|**	**95% Conf. Interval**	
						
Gender	7.284	7.340	1.970	0.049	1.010	52.503
Age at baseline	0.945	0.051	-1.050	0.293	0.851	1.050
Adiposity	1.431	0.511	1.000	0.316	0.711	2.881
Muscle function	0.117	0.118	-2.120	0.034	0.016	0.848
Cardio-metabolic function	0.521	0.194	-1.750	0.080	0.251	1.081
Neuro-sensory function	0.380	0.223	-1.650	0.100	0.120	1.202
constant	0.079	0.286	-0.700	0.484	0.000	97.444
						
Model information						
						
Observations	626	R^2^	0.50			
p Model	0.000					
Log likelihood	35.05					
						

**Table 4:** Emotional vitality baseline model predictions.

**Emotional vitality**	**Coef.**	**Std. Err.**	**z**	**P>|z|**	**95% Conf. Interval**	
						
Gender	0.839	0.346	-0.430	0.671	0.374	1.883
Age at baseline	1.018	0.013	1.400	0.161	0.993	1.044
Adiposity	0.974	0.163	-0.160	0.876	0.702	1.352
Muscle function	1.107	0.295	0.380	0.701	0.658	1.865
Cardio-metabolic function	0.363	0.176	-2.090	0.037	0.140	0.940
Neuro-sensory function	0.830	0.202	-0.770	0.444	0.514	1.338
constant	3.294	2.756	1.420	0.154	0.639	1.697
						
Model information						
						
Observations	623	R^2^	0.175			
p Model	0.000					
Log likelihood	32.610					
						

**Table 5:** Self-rated health baseline model predictions.

**Self-rated health**	**Coef.**	**Std. Err.**	**z**	**P>|z|**	**95% Conf. Interval**	
						
Gender	0.787	0.253	-0.740	0.456	0.420	1.477
Age at baseline	0.970	0.011	-2.770	0.006	0.949	0.991
Adiposity	1.128	0.140	0.980	0.330	0.885	1.438
Muscle function	0.549	0.129	-2.550	0.011	0.346	0.871
Cardio-metabolic function	0.481	0.108	-3.250	0.001	0.310	0.748
Neuro-sensory function	0.592	0.106	-2.930	0.003	0.418	0.841
constant	3.709	2.572	1.890	0.059	0.953	1.444
						
Model information						
						
Observations	623	R^2^	0.225			
p Model	0.000					
Log likelihood	-328,918					
						

**Table 6:** Relative latent factor contributions summary table.

	**Walking Speed**		**Dependency**		**Self-rated health**		**Emotional vitality**	
**Domain**	**T0**	**T9**	**T0**	**T9**	**T0**	**T9**	**T0**	**T9**
Gender		x	x			x		
Age at baseline	x	x		x	x	x		x
Adiposity	x	x		x		x		x
Muscle function	x	x	x	x	x	x		
Cardio-metabolic function	x			x	x		x	
Neuro-sensory function	x	x		x	x	x		
R^2^	0.78	N/A	0.50	N/A	0.23	N/A	0.17	N/A

In this graphic baseline measurements are represented as T0, and the nine-year follow-up measurement as T9.

Nine-year follow-up: Similar results were obtained at the nine-year follow-up, except for the finding that future *dependency* was not only predicted by muscle function, but by all four domains (Supplementary Table S9).

## DISCUSSION

There is a continued discussion in the literature as to what it means to age well, and terms vary from successful aging, active aging, positive aging, productive aging among others [29]. The aim of this study was to determine whether the phenotypic manifestation of aging can be measured parsimoniously. Exploratory factor analysis using the InCHIANTI database lead to the discovery of four domains: Neuro- sensory function, cardio-metabolic function, muscle function and adiposity. Logical relationships were found between the variables making up the factors of cardio-metabolic function, muscle function, and adiposity. Neuro-sensory function encompassed a compelling combination of measures of cognitive ability and sensory function, such as visual acuity and contrast sensitivity as well as other physiological factors. It was initially surprising that pulse pressure and insulin-like-growth-factor-1 (IGF-1) loaded on to this factor, but evidence from literature suggests strong and physiologically plausible relationships for this result. Previous longitudinal studies have found a relationship between higher pulse pressure and cognitive decline [30,31]. Baseline pulse pressure, for instance, has been associated with poorer executive ability and lower total cerebral volume and greater temporal horn ventricular volume after five to seven years of follow-up [32]. This is supported by similar findings showing prospective declines in learning, nonverbal memory, working memory, and a cognitive screening measure among participants with increasing levels of pulse pressure [31]. Insulin-like-growth-factor-1 on the other hand, has been shown to decline with age and precede cognitive decline [33]. Furthermore IGF-1 has been shown to play a major role in growth, aging, brain development and adult brain function [33], and specific associations have been made with reductions in fluid intelligence [34], and processing capacity [35].

When these domains were then used to determine the key aspects of successful aging, namely *walking speed*, *dependency risk*, *emotional vitality* and *self-rated health*, the directions of their contributions suggest that the domains are indeed useful. As summarized in table 6, both in the baseline and future walking speed model, high adiposity, and cardio-metabolic scores reduced walking speed, while high neuro-sensory and muscle function increased scores. In the baseline dependency model, only poor muscle function was predicted by dependency risk but, in the nine-year follow-up model, all domains became statistically relevant, with the strongest contributor being adiposity. These findings are in line with those of Diem *et. al.* who found that maintained independence among the oldest age was related to mobility and cognitive function [36].

The methods used here to develop a health score, and the outcomes we selected aid in avoiding the focus on ‘average tendencies’ within population subgroups, allow for heterogeneity and help shift the focus away from diseased and/or frail versus successful ager. Secondly, by carefully selecting the outcome variables we have avoided a focus on negative outcomes [37]. This also makes our model relevant to a wide range of the population by not limiting measures to those which are strictly related to frailty. In addition, by using only objective measurements, the influence of cultural differences may be reduced [38]. Lastly, by studying the aging individual in this way allows us to consider that successful aging may occur in the presence of (well managed) chronic disease [39] and recognises that aging and its influence does not begin at any predefined cut off.

In general, our results lend support to the two schools of thought on successful aging, specifically, the psychosocial school, which defines successful aging as a mental state and the biomedical school which suggests successful aging is avoidance of disease and disability [40]. Our model suggests physical aspects of aging can be predicted well in contrast to emotional resiliency and one’s health perspective.

### Limitations

What we have shown here is that combining variables in the form of scores representing different systems can determine two aspects of what we consider successful aging, namely walking speed and dependency risk, both of which can importantly be influenced by lifestyle change. However, we should carefully consider the context in which this model was developed. Variables were selected from a pre-existing database, with preference for those which were available at multiple time points. On the other hand, these variables were also selected due to their consistent relationship with the aging processes and were originally included in the database due to their possible relationship with disability [41]. Furthermore, we did not include early life factors, the impact of which is currently debated [42]. In addition, the factor analysis method should also be considered as the weightings of the specific variables and the composition of the factors may vary depending on the studied population. Our sample size also was limited because we chose to study a complete set of measurements. Lastly, we recognize that molecular metrics such as telomere length and methylation clock were not included as markers of biological ageing in the analysis. These measurements however are not normally done and cannot easily be added to typical blood panel chemistries. Furthermore, to date they are more theoretical instead of having practical use and for example, Haycock [43] elegantly demonstrated that telomere length remains controversial with respect to risk of cancer and non-neoplastic diseases [43].

## Conclusion

In a time of increasing longevity, reduced fertility rates, increased disease burden, as well as the availability of new and multiple alternative therapeutic opportunities, the ability to predict and measure the likelihood of an individual reaching old age, in a relatively good condition of health and wellbeing, is becoming progressively more important. From these considerations, our aim was to build a statistical that could help in building an objective operationalised definition of successful aging based on data collected in large longitudinal study performed in a representative population. Our work clearly shows that combining complex measurements allow the prediction of future health outcomes within the domain of successful aging. Our results show that parts of the aging trajectory can be determined from a body systems approach while others, specifically the components of healthy aging that are more subjective, cannot. Future research could focus on improving this scale, or aspects of this scale within aspects of it such that we can predict the likelihood of maintained health, ability and emotional wellbeing into old age.

## METHODS

### Study Design and participants

The data analysis was performed in the InCHIANTI database. InCHIANTI is a cohort survey that was designed and conducted to study risk factors and mechanisms of mobility loss in late life [41]. The initial data collection for this study began in September 1998 and the first phase was completed in 2000. Data collection continued thereafter every three years. For this study we used data collected at baseline (1998-2000) and in the three (2001 -2003), six (2004 -2006) and nine-year (2007 -2008) follow-up, which was concluded in 2009. Given the wide range of variables collected as well as the long follow-up, this cohort represents an extraordinary source for exploring factors associated with successful aging. A detailed description of the InCHIANTI cohort study can be found elsewhere [41]. In short, 1453 adults, aged 20 and over were randomly selected from the population registries in two towns in the Chianti countryside of Tuscany, Italy Greve in Chianti and Bagno a Ripoli, which represented 94% of the eligible population [44]. The study was approved by the Italian National Institute of Research and Care of Aging ethical committee and complies with the Declaration of Helsinki [44]. The InCHIANTI study collected data on physical function, cognitive function, social status, dietary habits, psychological status, laboratory parameters, disease history, family history and socioeconomic status [41]. All analyse was performed in Stata 14.2 [45].

### Variable selection

To select a putative list of variables that, at least in principle, could be potentially included as healthy aging indicators in variable clusters, we first identified variables from the InCHIANTI dataset that had been previously associated with aging and functional decline and had been included in other models of aging and/ or allostatic load, including but not limited to the HAP. Part of this search entailed examining the models which were recently included in a review by Mount *et. al*. [6] as well as looking at more recently developed models [28,46–49]. The final selection (Table 1) was based on expert opinion by the research team. Correlation analysis was performed to remove redundant variables.

A total number of 1453 of subjects were included in our analysis. Of these, 44% were male and 66% were female. Females were on average 69 years, and males 67 years at baseline and age ranged from 23 to 97 years and 21 to 102 years for males and females respectively. A complete data set was available for 506 observations, which were subsequently included in the EFA, in order to avoid techniques such as multiple imputation.

Additionally, multivariate regression analyses were performed to explore if the predictive value and individual contribution of the four health domains at baseline was similar after nine-year follow-up. These tables are presented in the addendum (Table S6-S9).

### Exploratory factor analysis

As a first step, to investigate the factor (domain) structure of the InCHIANTI dataset, we performed an exploratory factor analysis (EFA). We started by investigating multivariate normality using the Doornik-Hansen test [50] and the distributions of selected variables were explored by using histograms. Factorability of the data set was examined using an anti-image correlation matrix [51] [52]. Values of 0.3 or higher were considered appropriate to be included in the EFA. EFA was then carried out using the principle axis factoring method with standardised variables. Variables were standardised using the variable value divided by maximum minus the minimum value method [53]. If variables had a loading of (-) 0.3 or higher, on at least one factor they were retained in the model [54,55]. In the construction of these domains, the factor loading was carefully examined in the case of cross loadings. When variables had similar loadings on two factors, the variable factor was determined by logical relationships. In the case of HOMA, although it had a lower loading onto cardio-metabolic function, it was assigned to this factor as a result of testing both factor constructions. When assigned to the muscle function domain, it strongly reduced the reliability of the factor (0.65 to 0.56), while its addition to cardio-metabolic function had limited impact.

The resulting latent factors from the EFA were retained in the model based on the results of Kaisers criteria (eigen value greater than 1), as well as visual inspection of a scree plot (the number of factors to be retained in the solution is the number of factors which come before the elbow or levelling off of the curve) [55]. In order to determine if the latent factors were correlated both varimax and promax rotations were performed on the resulting factor structure [55]. Secondly, a correlation analysis was performed on the resulting factors to verify the existence of any correlations between factors. The presence of any correlations and or differences between rotation methods determines the appropriate rotation method. Factors which consisted of at least three variables were considered stable [55]. To determine the sampling adequacy of the dataset, the Kaiser-Meyer-Olkin (KMO) was applied, as a rule of thumb this value should be greater than 0.6 [56]. As a last step in the factor analysis, factor scores were then calculated using the predict function, a regression method in Stata, which were then used in further analysis.

### Reliability testing

Internal consistency and reliability were examined using Cronbach’s alpha for each of the extracted latent factors. Values greater than 0.9 were considered excellent, 0.8-0.9 good, 0.7-0.8 acceptable, 0.6-0.7 adequate, 0.5-0.6 poor and less than 0.5 as unacceptable.

### Factoring scoring

Factor scoring coefficients were derived from the discovered latent factors using a regression method. The weights of the individual variables were then multiplied by the standardised measurements of individual participants to determine individual variable scores. These scores were then added to give an overall score to each of the individual latent variables.

### Prediction models

Multivariate (logistic) regression analyses were used to determine the predictive value of the discovered factors (i.e. neuro-sensory function, muscle function, adiposity and cardio-metabolic function) on the key aspects of healthy aging; self-rated health, walking speed, emotional vitality, and dependency at baseline. Model fit was examined by using R^2^ in linear regression models and McKelvey and Zavoina's R^2^ in logistic models [57]. In the analysis age at baseline and gender used as covariates.

Mixed effect regression and mixed effect logistic models were used to predict the dependent variables self-rated health, walking speed, emotional vitality, and dependency at the nine-year follow-up. Models were adjusted for baseline age and gender. To do this we calculated factor scores for each of the time points. If a variable was not available at a specific follow-up point, it was substituted for the value at the most recent follow-up moment. Once the factor scores were calculated, as previously described, they were entered into the model as independent variables. In addition, a new variable, time point, which identified the factor scores at each time point, was included in the model as an independent variable.

***Walking speed.*** Walking speed (m/s) was based on a 400m walking test. If the participants were not able to complete the test, the estimated 400m walking speed (m/s) was used.

***Dependency.*** Participants were considered having disability if they had any need for help in performing Activities of Daily Living (ADL’s), reflecting the lack of ability to perform the eating, bathing, dressing, toileting, transferring and maintaining continence unaided [58].

***Self-rated health.*** Participants were considered as having poor self-rated health if they state that they health was very-poor, poor or fair and to have good self-rated health if they self-reported that they health was good and very good.

***Emotional vitality.*** Emotional vitality scores were generated following the method described by Penninx et. al. 2000 [13], with one exception. We had no complete measure of anxiety and therefore substituted the anxiety sub-score with the item from the CES-D questionnaire “During the past week, I felt fearful.” Participants were given a score of zero if they scored more than one on this question, indicating they felt fearful more than rarely in the past week. Participants were considered vital if they if they passed all items (i.e. a score of four) but were otherwise considered not-emotionally vital.

## SUPPLEMENTARY MATERIAL

Supplementary Figure

Supplementary Tables
