# Supplementary material for: Measuring successful aging: an exploratory factor analysis of the InCHIANTI Study into different health domains
Source: Aging (Albany NY). 2019 May 24;11(10):3023–40. doi: 10.18632/aging.101957 (PMC6555461; doi:10.18632/aging.101957)
Supplement: Supplementary Figure [file aging-11-101957-s001.pdf]

Supplementary Tables

Table S1. Orthogonal (Varimax) rotated factor loadings.

Variable	Factor 1	Factor 2	Factor 3	Factor 4	Uniqueness
Adiponectin		-0.3068			0.8279
Fat area at 66% tibia length			-0.7239		0.4144
Muscle area at 66% tibia length		0.4575			0.6889
Muscle density	0.4311	-0.3258			0.6768
TNFA-Receptor 2	-0.3755			-0.3793	0.6192
HOMA		0.5036			0.6636
Blood glucose		0.3533		-0.3170	0.7463
Creatinine			-0.5412		0.6918
Red cell distribution width	-0.3009				0.8993
Pulse Pressure	-0.4823				0.7458
Waist to hip ratio		0.3716	0.5119		0.5632
EPESE perform walking sub-score	0.3797			0.4470	0.6374
EPESE perform chair sub-score	0.4218			0.4348	0.5802
EPESE perform Balance sub-score	0.3433			0.4976	0.6092
**Coordination score**					0.9651
Coordination speed	0.7289				0.4397
Comorbidity score	-0.3855				0.7658
Muscle power lower extension max R side	0.6503		0.4477		0.3072
Trail making test B	-0.7464				0.4265
Years of education	0.6935				0.5005
**Hearing difficulty**					0.8857
IL6				-0.4217	0.7674
**CRP**					0.8350
IL1RA		0.4684			0.7427
**Cortisol: DHEAS ratio**					0.9549
**Ankle-brachial index**					0.9402
Cortical bone mass density	0.3487				0.8337
HDL cholesterol		-0.4921	-0.3040		0.6506
TIGF1	0.4341				0.8022
Olfactory score	0.4361				0.7618
Sensory score	0.6540				0.5171
**Social interaction score**					0.8937
Handgrip strength	0.5651		0.4749		0.4041
BMI		0.7464			0.3811
Visual acuity	0.5307				0.6950
Contrast sensitivity	0.5213				0.6863
MMSE score	0.6482				0.5785

Table S2. Regression generated scoring coefficients. Neuro-sensory.

Neuro-sensory coefficients	
Pulse pressure	-0.074
Coordination score	0.208
Trail making test B	-0.238
Years of Education	0.147
TIGF1	0.064
Olfactory score	0.075
Sensory score	0.147
Visual acuity (near Snellen)	0.123
Contrast sensitivity	0.123
MMSE score	0.143

Table S3. Regression generated scoring coefficients. Cardio-metabolic function.

Cardio-metabolic function coefficients	
TNFA- receptor 2	-0.103
HOMA index	-0.053
Blood glucose (mg/dl)	-0.051
z2PXSPSB	0.281
z2PXSPSC	0.234
z2PXSPSW	0.351
IL6	-0.119
CRP	-0.117

Table S4. Regression generated scoring coefficients. Muscle function.

Muscle Function	
Serum creatinine (reciprocal)	0.084
Waist to hip ratio)	0.069
Muscle power lower ext. max R side(watts)	0.463
Handgrip strength	0.435

Table S5. Regression generated scoring coefficients. Adiposity.

Adiposity coefficients	
Muscle area at 66% tibia length	0.129
Muscle density	-0.153
IL1RA	0.111
HDL	-0.111
BMI	0.564
Fat area at 66% tibia length	0.123

Table S6. Mixed effect model for walking speed.

Walking speed	Coef.	Std. Err.	z	P>|z|	95% Conf. Interval	
Age	-0.012	0.000	-33.290	0.000	-0.012	-0.011
Gender	0.117	0.011	10.470	0.000	0.095	0.139
Neuro-sensory function	0.005	0.001	6.740	0.000	0.004	0.007
Cardio-metabolic function	-0.001	0.001	-1.310	0.189	-0.003	0.001
Muscle function	0.001	0.000	9.150	0.000	0.001	0.002
Adiposity	-0.050	0.013	-3.840	0.000	-0.076	-0.025
Constant	1.953	0.023	84.690	0.000	1.908	1.998
**Random-effects Parameter var (Residual)**						
**Estimate**	0.033		**Wald chi2 (6)**		1537.030	
**Std. Err.**	0.001		**Log likelihood**		318.082	
**95% Conf. Interval**	0.030	0.035	**Number of obs.**		1081	
			**Prob> chi2 >**		0.000	

Table S7. Mixed effect model for emotional vitality.

Emotional vitality	Coef.	Std. Err.	z	P>|z|	95% Conf. Interval	
Age	0.005	0.001	7.210	0.000	0.004	0.006
Gender	-0.042	0.022	-1.880	0.060	-0.085	0.002
Neuro-sensory function	-0.001	0.002	-0.580	0.564	-0.004	0.002
Cardio-metabolic function	-0.002	0.002	-0.850	0.398	-0.006	0.002
Muscle function	0.000	0.000	-0.800	0.425	-0.001	0.000
Adiposity	-0.066	0.024	-2.770	0.006	-0.113	-0.020
Constant	0.533	0.047	11.440	0.000	0.442	0.624
**Random-effects Parameter var (Residual)**						
**Estimate**	0.389		**Wald chi2 (6)**		64.060	
**Std. Err.**	0.008		**Log likelihood**		-608.974	
**95% Conf. Interval**	0.374	0.404	**Number of obs.**		1285	
			**Prob> chi2 >**		0.000	

Table S8. Mixed effect model for self-rated health.

Self-rated health	Coef.	Std. Err.	z	P>|z|	95% Conf. Interval	
Age	0.004	0.001	5.200	0.000	0.002	0.005
Gender	-0.103	0.024	-4.310	0.000	-0.149	-0.056
Neuro-sensory function	-0.004	0.002	-2.410	0.016	-0.007	-0.001
Cardio-metabolic function	-0.003	0.002	-1.330	0.184	-0.007	0.001
Muscle function	-0.001	0.000	-2.880	0.004	-0.002	0.000
Adiposity	0.069	0.026	2.700	0.007	0.019	0.120
Constant	0.095	0.050	1.900	0.058	-0.003	0.194
**Random-effects Parameter var (Residual)**						
**Estimate**	0.422		**Wald chi2 (6)**		727.050	
**Std. Err.**	0.008		**Log likelihood**		-727.050	
**95% Conf. Interval**	0.406	0.438	**Number of obs.**		1309	
			**Prob> chi2 >**		0.000	

Table S9. Mixed effect model for dependency.

Dependency	Coef.	Std. Err.	z	P>|z|	95% Conf. Interval	
Age	0.002	0.000	5.630	0.000	0.001	0.002
Gender	-0.006	0.010	-0.560	0.577	-0.025	0.014
Neuro-sensory function	-0.002	0.001	-3.440	0.001	-0.004	-0.001
Cardio-metabolic function	-0.003	0.001	-2.620	0.009	-0.004	-0.001
Muscle function	-0.001	0.000	-3.360	0.001	-0.001	0.000
Adiposity	0.027	0.010	2.620	0.009	0.007	0.048
Constant	-0.050	0.021	-2.360	0.018	-0.092	-0.009
**Random-effects Parameter var (sd Residual)**						
**Estimate**	0.181			**Wald chi2 (6)**	77.650	
**Std. Err.**	0.003			**Log likelihood**	393.619	
**95% Conf. Interval**	0.175	0.188		**Number of obs.**	1361	
				**Prob> chi2 >**	0.000	
