# Supplementary material for: Measuring successful aging: an exploratory factor analysis of the InCHIANTI Study into different health domains
Source: Aging (Albany NY). 2019 May 24;11(10):3023–40. doi: 10.18632/aging.101957 (PMC6555461; doi:10.18632/aging.101957)
Supplement: Supplementary Tables [file aging-11-101957-s002.pdf]

SUPPLEMENTARY MATERIAL

Supplementary Figures

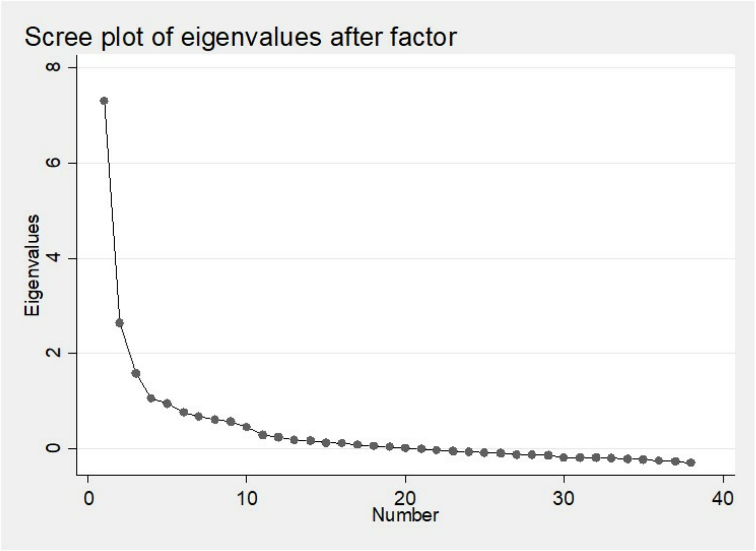

Figure S1. Scree plot: Scree plot demonstrating an elbowing of the curve at factor number three or four.
